# Retraction for: Cationic solid lipid nanoparticles loaded by integrin β1 plasmid DNA attenuates IL-1β-induced apoptosis of chondrocyte

**DOI:** 10.18632/aging.205326

**Published:** 2023-11-15

**Authors:** Yuejiang Zhao, Hanwen Chen, Lu Wang, Zhiyuan Guo, Shijie Liu, Simin Luo

**Affiliations:** 1 The First Department of Orthopedics, Cangzhou Central Hospital, Cangzhou, Hebei Province, China; 2 Department of Joint Surgery, Xingtan Hospital Affiliated to Shunde Hospital of Southern Medical University, Foshan, Guangdong Province, China; 3 Department of Bone and Joint Surgery, The First Affiliated Hospital, Jinan University, Guangzhou, Guangdong Province, China

**Keywords:** osteoarthritis, integrin β1, cationic solid lipid nanoparticles, chondrocytes

**This article has been retracted:** Aging has completed its investigation of this paper after receiving alerts from the PubPeer platform regarding multiple inconsistences in **Figure 4B** as well as duplication of results from a separately published paper from a different author group [1]. We confirmed that **Figure 2A**, “Gel retardation assay of naked pDNA and SLNs/pDNA complexes with the different mass ratio,” duplicates Figure 1D, "Gel retardation assay of naked siRNA and MWNT/Sor/siRNA complexes with the different N/P ratio," published by other authors [1]. We also found internal duplications of results with BrdU- and DAPI-stained cells from other experimental groups presented in **Figure 4B** as well as inconsistency between the BrdU and DAPI images from the same experimental group and, as a result, a disconnect in the merged images. The authors notified us that they see the problems in the paper and all authors agreed that the article should be retracted.

## References

[1] Wen Z, Feng Y, Hu Y, Lian L, Huang H, Guo L, Chen S, Yang Q, Zhang M, Wan L, Xu K, Degejirifu, Yan X. Multiwalled carbon nanotubes co-delivering sorafenib and epidermal growth factor receptor siRNA enhanced tumor-suppressing effect on liver cancer. Aging (Albany NY). 2021; 13:1872–82. 10.18632/aging.103905 33440348 PMC7880368

